# Cumulative metabolic stress (microfilarial infection + moult) constrains the expression of carotenoid-based honest signals in breeding male village weavers (*Ploceus cucullatus*) of Amurum Forest Reserve, Nigeria

**DOI:** 10.1371/journal.pone.0350806

**Published:** 2026-06-05

**Authors:** Felix A. Andong, Olufemi Olasoji, Abdifatah Ahmed A. Afyare, Ezekiel S. Mayowa, Praise O. Nwanozie, Emmanuel E. Osayi, Ruth A. Agyo, Vincent C. Ejere

**Affiliations:** 1 Department of Zoology and Environmental Biology, Faculty of Biological Sciences, University of Nigeria, Nsukka, Enugu, Nigeria; 2 Faculty of Natural Sciences, AP Leventis Ornithological Research Institute, University of Jos, Jos, Plateau, Nigeria; 3 Department of Social Science, Faculty of Economics and Management Science, Salaam University, Mogadishu, Somalia; 4 Department of Plant Science and Biotechnology, Faculty of Biological Sciences, University of Nigeria, Nsukka, Enugu, Nigeria; 5 Department of Public Veterinary, Public Health and Preventive Medicine, College of Veterinary Medicine, Joseph Sarwuan Tarka University, Makurdi, Nigeria; Instituto Leonidas e Maria Deane / Fundacao Oswaldo Cruz, BRAZIL

## Abstract

In wild birds, the breeding season involves a convergence of metabolically demanding life-history stages, including reproduction, moult, and immune defense. We investigated the relationships between microfilarial infection, moult, redox homeostasis, and plumage quality in breeding male village weavers (Ploceus cucullatus) at the Amurum Forest Reserve, Nigeria. We compared four groups (n = 148 total) sampled within 3 mins post-capture: infected-moulting (IM), infected-non-moulting (IN), non-infected-moulting (NM), and non-infected-non-moulting (NN). Physiological condition was assessed using the erythrocyte glutathione ratio (GSH:GSSG) and circulating glucose, while plumage reflectance traits were integrated into a composite quality axis (PC1). Microfilarial infections were present in 52.0% (n = 77) of individuals; mean parasite intensities were 6.13 ± 0.35 mf/µL (IN) and 6.45 ± 0.41 mf/µL (IM). Physiological indices varied strongly across groups. The GSH:GSSG ratio was reduced in infected birds, indicating altered redox balance (rs = −0.65). Circulating glucose was highest in the infected non-moulting group (IN) but substantially reduced in the infected moulting group (IM). Across physiological and ornamental traits, individuals experiencing both infection and moult (IM group) exhibited the strongest reductions relative to all other groups. However, this pattern reflects a statistically supported Infection × Moult interaction, rather than an untested synergistic or non-linear effect, as evidenced by significant IN vs. IM contrasts in glucose (Table 3; z = 33.43, *P* < 0.0001, d = 6.10) and plumage quality. This interaction was associated with reduced plumage hue and lower integrated signal quality. Our results suggest that microfilarial infection and moult impose overlapping physiological demands that constrain metabolic regulation and the expression of carotenoid-based ornaments. Intracellular redox balance emerges as a potential mechanistic link between physiological state and ornamental expression, supporting the hypothesis that village weaver plumage reflects variation in sustained physiological condition under natural ecological stress.

## Introduction

In wild birds, the breeding period represents a convergence of metabolically demanding life history stages. Although parasitism and moult are often studied independently, both processes place substantial strain on the antioxidant system and compete for limited physiological resources [1–3]. As a result, individuals experiencing infection, moult or both frequently exhibit reduced body condition, impaired feather replacement and altered stress biomarkers [4–6]. The quality of plumage reflectance in parasite infected hosts is known to influence behavioral outcomes during the breeding season [7]. Indeed, parasitism during moult is expected to increase physiological stress by altering nutrient allocation between maintenance and immune defense [8,9]. As such, blood parasites such as microfilarial infection are known to influence indicators of body condition in birds [5,10], particularly when they occur alongside moult, where their combined effects can generate resource allocation trade-offs that may compromise condition dependent traits [11,12]. Such interactions may increase reactive oxygen species (ROS) and disrupt antioxidant enzyme activity [13–15], ultimately reducing the quality of plumage signals that reflect physiological and nutritional state [16,17].

Previous studies have often relied on single markers of oxidative damage, e.g., malondialdehyde (MDA) to assess oxidative stress; however, such approaches may not adequately capture the combined effects of moult and parasitic infection [18–21]. These approaches can be confounded by diet and fluctuating lipid profiles, which complicates physiological interpretation [18,22]. It is often unclear whether observed values reflect inherent antioxidant capacity or a compensatory response to ongoing oxidative stress [19,20]. The ratio of reduced to oxidized glutathione (GSH:GSSG) could provide a more integrative assessment by capturing both antioxidant availability and consumption. This ratio serves as a sensitive indicator of intracellular oxidative status and highlights the central role of glutathione in mediating life history trade-offs of moulting birds [15,23–27]. Moult is an energetically demanding process that increases metabolic rate by approximately 28% and places additional pressure on redox homeostasis in infected birds [28–30].

This study is motivated by the observation that breeding male village weavers (*Ploceus cucullatus*) provide an ideal system to examine these constraints because individuals infected with microfilarial infection often continue to moult despite having already attained full breeding plumage [10,31]. While the oxidative costs of parasitism and moult are well documented individually [14,15,28,30,32], their combined effects in free living, breeding individuals remain poorly resolved [33–35]. In this context, the GSH: GSSG ratio offers a powerful biomarker for linking intracellular oxidative state to the phenotypic expression of sexual signals [36,37]. By integrating the glutathione redox system with high-resolution plumage reflectance spectroscopy, we provide mechanistic insight into how intracellular oxidative state is associated with the expression of condition-dependent ornaments.

Our primary objectives were to: (i) evaluate whether the interaction between infection and moult is characterized by a synergistic physiological cost using the GSH:GSSG ratio and circulating glucose as indicators of redox status and metabolic demand [38]; (ii) determine if this metabolic burden is intensity dependent by exploring the correlation between infection severity and physiological markers; (iii) identify specific physiological differences associated with infection during the peak energetic demands of feather synthesis by comparing NM vs. IM groups; and (iv) assess the relationships between parasite presence and plumage reflectance traits brightness, hue and chroma to link internal metabolic condition to external ornamental expression [39,40].

We hypothesize that concurrent infection and moult induce a synergistic, non-linear decline in physiological condition, reflected in significantly lower GSH: GSSG ratios and reduced circulating glucose in the IM group compared to the NM, IN and NN groups. We further hypothesize that this cumulative physiological stress will reduce the quality of condition dependent plumage traits. These traits are expected to correlate most strongly with GSH: GSSG ratios in moulting birds, providing a potential indicator of the costs of signal maintenance when breeding males cannot buffer simultaneous metabolic demands. Accordingly, this study empirically tests the metabolic constraint hypothesis, which posits that simultaneous immune activation and rapid keratin synthesis may reduce redox buffering capacity, thereby constraining the expression of sexual signals in birds.

## Materials and methods

### Ethical approval statement

This study received formal approval from the scientific committee of the A.P. Leventis Ornithological Research Institute (APLORI) at the University of Jos. While institutional oversight was maintained, all fieldwork activities and site access were conducted through local arrangements and established protocols within the study area to ensure adherence to both scientific and ethical guidelines.

### Study area/ bird trapping

This study was conducted from June to August 2023 at the A.P. Leventis Ornithological Research Institute’s (APLORI) Amurum Forest Reserve (09°53′ N, 08°59′ E; Plateau State, Nigeria). Fieldwork coincided with the peak breeding season of the village weaver, which typically occurs during the rainy months of May to October to take advantage of increased food and nesting material availability [41,42]. Breeding male birds were captured between 06:30 and 10:30 h using mist nets (12 m x 2.6 m, 16 mm mesh size) placed near active breeding sites (away from the constant effort sites) of APLORI in Amurum Forest Reserve. Ringing was guided by the APLORI ringing guidelines. To account for the temporal dynamics in host activity and parasite intensity identified in our previous work, sampling was categorized into three intervals: early morning (6:30–7:59 a.m.), mid-morning (8:00–9:29 a.m.), and late morning (9:30–10:30 a.m.) [43]. Upon capture, each individual was fitted with a uniquely numbered aluminum ring to facilitate identification and prevent pseudoreplication through repeated sampling. Hence all bird handling and ringing procedures strictly adhered to the APLORI ringing guidelines, which are aligned with international standards for avian welfare and data integrity. Any bird recaptured during the study period was identified by its ring number and excluded from further sampling, ensuring that our dataset consists of unique individuals. On the other hand, the structured sampling approach allowed us to include time period as a covariate in our final models. This ensured that the detected physiological effect was not confounded by diurnal fluctuations in bird condition, capture bias, or the known sensitivity of circulating glucose to daily feeding and activity cycles.

### Phenotypic features of adult breeding males

For this study, only adult males possessing high-quality breeding features were trapped and sampled. The village weaver is a gregarious, dimorphic and polygamous passerine. Breeding males undergo a moult to acquire a distinct reproductive phenotype characterized by bright nuptial plumage: a black crown, face, chin, and throat; a dark chestnut nape and neck sides; and a mottled black and yellowish back forming a V-shape. Additionally, they display a yellow rump, an olive tail and more reflective yellow underparts. During breeding, the eyes become more orange-red, the bill darkens, and the legs and feet turn brownish [44].

### Assessment of moult/ feather collection

Filarial infections and moult were assessed exclusively in adult males that had attained full breeding plumage. Individuals were categorized as moulting based on the presence of actively growing or replacing feathers, while those without such plumage activity were classified as non-moulting. From each breeding male, three primary plumage patches were sampled: (i) a cluster of carotenoid based yellow breast feathers, (ii) eumelanin based black crown feathers, and (iii) phaeomelanin rich brown nape feathers. Additionally, the right outermost (6th) tail feather was collected as a standardized measure of structural and melanin based signal quality; although this feather displays a yellowish olive hue, it serves as a contrast to the pure carotenoid signals of the breast to assess whether physiological stress impacts rectrix integrity differently than high cost body pigments [45].

For each identified plumage patch, approximately 5 contour feathers were gently removed using fine tipped stainless steel forceps to ensure precision and minimize follicle trauma. For the breast, crown and nape, feathers were plucked from the center of the respective color patches. The right outermost (6^th^) tail feather was grasped firmly at the base of the calamus and removed with a single, swift motion in the direction of growth to preserve the entire structural signal for high resolution reflectance spectroscopy. Immediately following collection, all feather samples were placed into individual, lightproof paper envelopes to prevent photodegradation of the sensitive carotenoid and melanin pigments. Each envelope was clearly labeled with the unique bird identification number, date, and site location. To maintain sample integrity and prevent fungal growth or structural warping, the envelopes were stored in a climate controlled environment with low humidity and kept in total darkness until laboratory processing.

### Blood collection

Following physical assessment, approximately 80 µL of whole blood was collected from the brachial vein for assessing circulating glucose and the GSH:GSSG ratio. Blood sampling was completed within 3 mins post-capture to minimize capture-induced physiological alteration and ensure that measured values reflected baseline physiological state rather than handling stress responses. Circulating glucose was used as a real-time proxy for metabolic demand and energy mobilization. The same 3 mins post-capture window was applied to ensure comparability of glucose and erythrocyte glutathione measurements under standardized conditions [46–48]. Immediately following collection, thin blood smears were prepared for microfilaria infection screening, while remaining blood was stored in heparinized hematocrit tubes for laboratory analysis. Samples were used for infection screening via buffy coat techniques and for erythrocyte glutathione and redox assays. To preserve intracellular redox integrity, tubes were immediately sealed and stored on ice (4°C) for transport to the laboratory.

### Circulating glucose assay

Glucose was measured immediately after collection using a CentriVet® point-of-care (POC) device. Glucose concentration was determined by inserting test strips into the device, with the sample applied to the strip tip. The enzymatic reaction between glucose and strip reagents generated an electrical signal, which was converted into glucose concentration values within a range of 10–600 mg/dL (CentriVet GK 2016).

### Microfilarial infection screening

Heparinized capillary tubes were centrifuged at 10,000 × g for 10 minutes to separate blood components and obtain the buffy coat. Microfilarial infections were identified from buffy coat preparations and thin blood smears following established protocols [49,50]. Slides were examined under oil immersion using a Nikon microscope, and parasites were identified based on standard morphological criteria [51]. Parasite intensity was quantified as microfilariae per microlitre of blood (mf/µL) using combined buffy coat and smear-based counts, representing absolute parasite load rather than prevalence or percentage infection. The packed erythrocyte fraction was then isolated for downstream biochemical analyses.

### Erythrocyte glutathione/ redox status assay

Glutathione was quantified to assess antioxidant capacity and the GSH: GSSG ratio using the enzymatic recycling method of described in Griffith [52], with modifications [53,54]. Erythrocytes were diluted (1:10 w/v) and homogenized in 0.01 M phosphate-buffered saline (PBS) containing 0.02 M EDTA, with all steps performed on ice. The homogenate was deproteinized by vortexing with an equal volume of 10% (w/v) trichloroacetic acid (TCA) (3 × 5 s within 15 minutes), while kept refrigerated and protected from light. Samples were then centrifuged at 10,000 × g for 10 minutes at 4°C, and the supernatant was collected. Glutathione levels were measured spectrophotometrically (Thermo Fisher Scientific® NanoDrop 2000, Waltham, MA, USA) based on DTNB reduction by NADPH in the presence of glutathione reductase (GR). Total glutathione (tGSH) was assayed in a reaction medium containing 100 mM KPi (pH 7.0), 1 mM EDTA, 0.1 mM NADPH, 0.1 mM DTNB, and 0.05 U/mL GR, with absorbance read at 412 nm against a 0.031–1 mM standard curve. For oxidized glutathione (GSSG), aliquots were adjusted to pH 7.5 with 6 N NaOH and incubated with 2-vinylpyridine in the dark to mask GSH, followed by centrifugation (10,000 × g, 10 minutes). GSSG was then quantified using a reaction medium containing 0.3 U/mL GR. Concentrations of tGSH and GSSG were expressed as µmol/g erythrocyte pellet. Reduced glutathione was calculated as GSH = tGSH − (2 × GSSG), and the GSH: GSSG ratio was used as an index of intracellular redox status across study groups.

### Plumage reflectance traits

Plumage reflectance was quantified using a high resolution miniature fiber optic spectrophotometer (Ocean Optics S2000) coupled to a pulsed xenon light source [55,56]. Measurements were conducted in a darkened room to eliminate ambient light interference. For each of the four sampled patches (carotenoid based breast, eumelanin based crown, phaeomelanin rich brown nape, and the 6th tail feather), feathers were arranged in a standardized overlapping mat on a matte black non reflective card. Reflectance was measured from a standardized position on the dorsal side of the feathers. For the breast and nape patches, measurements were taken approximately 5 mm inward from the apex of the feather on both the inner and outer vanes. Light reached the feather surface at a 90º angle, while the sampling optic was placed at a 45º angle to capture diffuse reflectance. The instrument was calibrated every 15 minutes against a WS 2 diffuse white standard and a dark standard. Afterwards, four measurements (two from each vane) were averaged to obtain a single representative spectral curve. The brightness, chroma and hue of feather coloration were analyzed based on the reflectance data. The values were calculated using the following spectral ranges: UV 300–400 nm (to account for the avian tetrachromatic visual system and to capture structural signal components that are invisible to the human eye but ecologically relevant for mate assessment); blue 400–475 nm; green 475–550 nm; yellow 550–625 nm; and red 625–700 nm. Following the protocols described by Endler (1990), brightness was calculated as the sum of relative reflectance across the entire spectral range of 300–700 nm, while chroma and hue were derived as follows:


$$\begin{array}{c} Brightness=\;\sum_{\text{3}\text{0}\text{0}}^{\text{7}\text{0}\text{0}}Ri \end{array}$$

$$\begin{array}{c} \text{Chroma }=\;{\left({\text{R}}_{\text{625}-\text{700}}\;-\;{\text{R}}_{\text{475}-\text{550}}\right)}^{\text{2}}\;+\;{\left({\text{R}}_{\text{550}-\text{625}}\;-\;{\text{R}}_{\text{400}-\text{475}}\right)}^{\text{2}} \end{array}$$

$$\begin{array}{c} \text{Hue }=\text{ arcsin }[({\text{R}}_{\text{550}-\text{625}}\;-\;{\text{R}}_{\text{400}-\text{475}})/\text{chroma}] \end{array}$$

### Data analyses

All statistical analyses were conducted in *R* version 4.3.1 [57]. To ensure comparability across variables measured on different scales, all continuous response variables, including physiological markers (GSH: GSSG ratio and circulating glucose) and ornamental traits (brightness, chroma, and hue), were Z-score standardized to a mean of 0 and a standard deviation of 1. This transformation facilitated the direct comparison of standardized effect sizes (beta coefficients) and served as a prerequisite for Principal Component Analysis (PCA). The PCA was used to integrate multiple reflectance traits into a single composite plumage quality axis (PC1), which accounted for 80.3% of the total variance; subsequent analyses utilized PC1 as the primary measure of ornamental quality.

Prior to modeling, variables were screened for outliers using Z-score thresholds where the absolute value of Z was greater than 3; as no extreme outliers were detected (*n* = 148), all observations were retained to preserve natural biological variation. Assumptions of normality and homoscedasticity were assessed using Shapiro–Wilk and Levene’s tests. Although deviations from normality were detected for the GSH: GSSG ratio (W = 0.91, *P* < 0.001), plumage quality (W = 0.95, *P* < 0.001), and glucose (W = 0.94, *P* < 0.001), model adequacy was ensured through visual inspection of residual diagnostic plots. To enhance the robustness of inference and address potential heteroscedasticity, all analyses were conducted using Robust Linear Models (RLM) based on M-estimation, complemented by heteroscedasticity-consistent (HC3) standard errors. Furthermore, Variance Inflation Factors (VIF) were calculated to confirm the absence of multicollinearity among predictors, with all VIF values remaining below 2.0.

To test the primary hypothesis that infection and moult impose synergistic physiological costs, we employed a Global Modeling approach with Type III sums of squares. This approach avoids the instabilities associated with automated variable selection by including all biologically relevant predictors a priori. Non-infected and non-moulting individuals (NN) served as the reference control group. Objective (i) was addressed by modeling the GSH: GSSG ratio and circulating glucose as response variables against fixed predictors of infection status and moulting status. Objective (ii) examined ornamental signaling by modeling PC1 against the same predictors. In all global models, sampling time (early, mid, and late morning) was retained as a fixed effect to control for potential diurnal variation and capture bias.

The effect sizes for model terms were quantified using partial eta squared (ηp^2^) [58], representing the proportion of variance explained by the group factors. To evaluate the magnitude of specific group differences, we calculated Cohen’s d [59], with values of 0.2, 0.5, and 0.8 interpreted as small, medium, and large effects, respectively. For statistically significant categorical effects (*P* < 0.05), post hoc pairwise comparisons were conducted using Tukey-adjusted linear contrasts. Finally, the relationship between microfilarial intensity and physiological markers was evaluated using Spearman’s rank correlations (r_s_), providing a robust assessment of intensity-dependent metabolic burdens. All statistical tests were evaluated at a significance threshold of 0.05.

## Results

A total of 148 breeding male village weavers were sampled across four physiological groups: healthy baseline (NN, *n* = 42), isolated moult (NM, *n* = 29), isolated infection (IN, *n* = 46), and synergistic stress (IM, *n* = 31). Within this study population, microfilarial infection was prevalent in 52% (*n* = 77) of the individuals, providing a robust distribution of parasitic burden across both the moulting and non-moulting cohorts. Specifically, microfilarial intensities were 6.13 ± 0.35 mf/µL in the isolated infection (IN) group and 6.45 ± 0.41 mf/µL in the synergistic stress (IM) group, indicating comparable parasite loads across infected individuals. These values represent absolute parasite density in blood (microfilariae per microlitre of blood) rather than proportional percentages. The descriptive physiological and plumage metrics varied significantly across these cohorts (Table 1). Microfilarial intensity was absent in the non-infected NN and NM groups (0.00 ± 0.00 mf/µL), while comparable parasite loads were maintained in the infected IN (6.13 ± 0.35 mf/µL) and IM (6.45 ± 0.41 mf/µL) groups. Physiological markers revealed distinct metabolic shifts. The GSH: GSSG ratio was highest in the NM group (2.11 ± 0.01), followed closely by the NN baseline (2.08 ± 0.05), however, the ratio was markedly depleted in the IN (0.55 ± 0.04) and IM (0.93 ± 0.02) groups. Circulating glucose (mg/dL) was highest in the infected non-moulting group (IN; 307.8 ± 2.8), but reduced to the lowest levels in the synergistic IM group (183.4 ± 4.1). Plumage traits remained most stable in the non-infected groups. Hue was similar between NN (55.1 ± 0.3°) and NM (55.5 ± 0.08°), but declined in IN (49.4 ± 0.3°) and IM (51.6 ± 0.3°) groups. Brightness was slightly higher in the healthy baseline (NN: 45.6 ± 0.6%; NM: 48.3 ± 1.36%) and infected IN group (58.1 ± 0.8%), but declined in the IM group (52.9 ± 0.9%). Chroma was lowest in the non-infected groups (NN: 0.39 ± 0.01; NM: 0.43 ± 0.00), but higher in the IN group (0.60 ± 0.01).

**Table 1:** Mean physiological and plumage metrics across intensity of infection and moult status. Indicating the descriptive statistics for the four groups. Non-infected and non-moulting (NN) represents the healthy baseline; non-infected and moulting (NM) represents the isolated effect of moulting; infected and non-moulting (IN) shows the isolated effect of blood parasites, while the infected and moulting (IM) represents the synergistic stressor interaction. These values indicate the mean physiological burden and morphological shifts observed when infection and moult co-occur in the village weavers (*n* = 148, total breeding males). Out of the total population sampled, microfilarial infection was present in 52.0% (n = 77) of breeding male village weavers, while parasite intensity values are reported as microfilariae per microlitre of blood (mf/µL), derived from buffy coat and smear-based enumeration.

Variable	NN *n* = 42 (28%)	NM *n* = 29 (20%)	IN *n* = 46 (31%)	IM *n* = 31(21%)
**Physiology**				
Microfilarial intensity (mf/µL)	0.00 ± 0.00	0.00 ± 0.00	6.13 ± 0.35	6.45 ± 0.41
GSH:GSSG Ratio	2.08 ± 0.05	2.11 ± 0.01	0.55 ± 0.04	0.93 ± 0.02
Glucose (mg/dL)	239.1 ± 3.2	249.3 ± 2.3	307.8 ± 2.8	183.4 ± 4.1
**Plumage Quality**				
Hue (º)	55.1 ± 0.30	55.5 ± 0.08	49.4 ± 0.30	51.6 ± 0.30
Brightness (%)	45.6 ± 0.60	48.3 ± 1.36	58.1 ± 0.80	52.9 ± 0.90
Chroma (purity)	0.39 ± 0.01	0.43 ± 0.00	0.60 ± 0.01	0.49 ± 0.01

**Fig 1:**
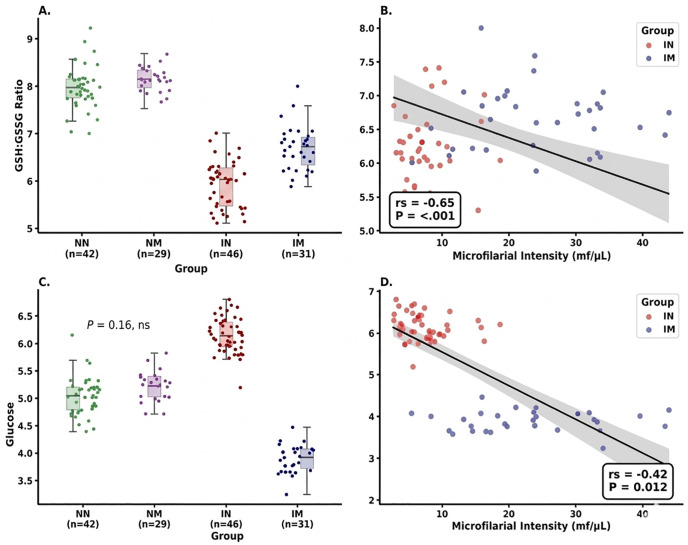
Physiological effects of microfilarial infection and moult in breeding male village weavers. **(A and C)** Box-and-jitter plots showing variation in physiological condition across experimental groups: non-infected non-moulting (NN, *n* = 42), non-infected moulting (NM, n = 29), infected non-moulting (IN, *n* = 46), and infected moulting (IM, *n* = 31). Panel A presents the erythrocyte GSH:GSSG ratio, while panel C presents circulating glucose (mg/dL). Group effects indicate significant differences in redox balance and metabolic state across infection and moult categories (*P* < 0.0001). **(B and D)** Scatterplots showing relationships between microfilarial intensity (mf/µL) and physiological variables in infected individuals (IN and IM groups only). Panel B shows a significant negative correlation between parasite load and GSH:GSSG ratio (rs = −0.65, *P* < 0.001), while panel D shows a significant negative relationship between parasite load and circulating glucose (rs = −0.42, *P* = 0.012). Shaded regions represent 95% confidence intervals from fitted regression models. All values are presented for 148 breeding males.

Conversely, the robust linear models (RLM) revealed that group status had a strong and significant effect on both physiological markers (Table 2; Fig 1A and C). For the GSH: GSSG ratio, group was a dominant predictor (F₃, ₁₄₂ = 312.44, *P* < 0.0001, ηp² = 0.86), whereas sampling time had no detectable effect (F₂, ₁₄₂ = 0.091, *P* = 0.91, ηp² < 0.01). A similar pattern was observed for glucose, with a strong group effect (F₃, ₁₄₂ = 428.15, *P* < 0.0001, ηp² = 0.89) and no effect of sampling time (F₂, ₁₄₂ = 0.128, *P* = 0.88, ηp² < 0.01). Pairwise contrasts from the RLM indicated significant differences among groups for both physiological variables (Table 3; Fig 1A and C). For the GSH: GSSG ratio, all comparisons were significant (*P* < 0.05), with the largest effect observed between NN vs. IN (d = 5.43), followed by NN vs. IM (d = 3.65), and IN vs. IM (d = 1.91). Microfilarial intensity was strongly negatively correlated with the GSH: GSSG ratio (rs = −0.65, *P* < 0.001), indicating reduced redox balance with increasing parasite burden (Fig 1B). For glucose, pairwise contrasts were also significant across major comparisons (*P* < 0.0001), with large effect sizes observed: NN vs. IN (d = 3.02), NN vs. IM (d = 3.11), and IN vs. IM (d = 6.10). Glucose levels were negatively correlated with microfilarial intensity (r_s_ = −0.42, *P* = 0.012), indicating a decline in circulating metabolic fuel with increasing parasite load (Fig 1D). Importantly, the Infection × Moult interaction was explicitly supported within the factorial model structure, with strong evidence of effect modification indicated by the pronounced divergence between infected non-moulting (IN) and infected moulting (IM) groups, and statistically supported by significant IN vs. IM contrasts for both glucose (d = 6.10, P < 0.0001) and GSH:GSSG ratio (d = 1.91, P < 0.001), hence confirming that infection effects differ depending on moult status within the birds.

**Table 2:** Analysis of deviance for physiological markers of redox homeostasis and metabolic fuel. Indicating the Robust Linear Model (RLM) evaluating the effects of experimental group (infection and moult status) and sampling time on the GSH: GSSG ratio (a marker of oxidative stress) and circulating glucose levels (a marker of metabolic fuel). The high F-values for the Group variable indicate that the physiological state of the village weavers is profoundly influenced by the overlap of stressors, while the non-significant *p*-values for time confirm that sampling intervals did not introduce systemic bias into the results.

Response Variable	Source	Df	*F*	*P*	Effect Size (ηp2)
GSH:GSSG Ratio	Group	3	312.44	< 0.0001	0.86
	Time	2	0.091	0.91	< 0.01
Glucose	Group	3	428.15	< 0.0001	0.89
	Time	2	0.128	0.88	< 0.01

**Table 3:** Comprehensive analysis of physiological markers in Village Weavers, including pairwise contrasts and intensity-dependent correlations. Indicating the standardized pairwise comparisons derived from the Robust Linear Model (RLM) evaluating differences between groups (NN: Non-infected; NM: Non-infected and Moulting; IN: Infected; IM: Infected and Moulting), followed by Spearman’s rank correlations (rs) assessing the intensity-dependence of microfilarial load on host physiology within the infected cohorts (in bold). Cohen’s d represents the standardized effect size for contrasts, while rs denotes the correlation coefficient for the intensity-dependent physiological burden.

Variable	Contrast Type	Specific Contrast	Estimate/ r_s_	Statistic (z)	*P*	Cohen’s d
GSH:GSSG	Healthy Baseline	NN vs. NM (Moult cost)	0.03	2.58	0.010	0.11
	Infection Effect	NN vs. IN (Primary Cost)	2.08	29.77	<.0001	5.43
	Isolating Cost	NM vs. IM (Infection in Moult)	1.33	18.22	<.0001	3.54
	Interaction	IN vs. IM	−0.72	−10.43	<.0001	1.91
	Cumulative Stress	NN vs. IM (Synergy)	1.36	19.97	<.0001	3.65
	**Intensity**	**Parasite Load (r**_**s**_ **)**	**−0.65**	**—**	**< 0.001**	**—**
Glucose	Healthy Baseline	NN vs. NM (Moult cost)	0.10	1.41	0.16	0.25
	Infection Effect	NN vs. IN (Primary Cost)	−1.17	−16.52	<.0001	3.02
	Isolating Cost	NM vs. IM (Infection in Moult)	1.07	15.64	<.0001	2.86
	Interaction	IN vs. IM	2.34	33.43	<.0001	6.10
	Cumulative Stress	NN vs. IM (Synergy)	1.17	17.05	<.0001	3.11
	**Intensity**	**Parasite Load (r**_**s**_ **)**	**−0.42**	**—**	**0.012**	**—**

**Fig 2:**
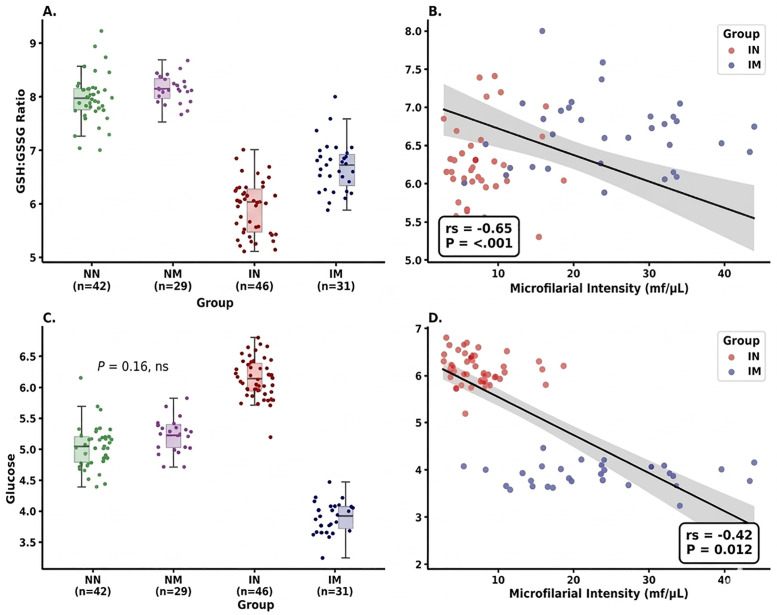
Integrated plumage quality (PC1) as a condition-dependent trait in village weavers. **(A)** Box-and-jitter plots showing variation in plumage quality (PC1 scores) across experimental groups. Significant differences were detected among groups (*P* < 0.0001), with non-infected individuals (NN and NM) generally exhibiting higher PC1 scores than infected groups (IN and IM). Brackets indicate pairwise comparisons from the RLM; asterisks (***) denote statistically significant differences, while “ns” indicates a non-significant contrast between healthy baseline groups. **(B)** Scatterplot illustrating the relationship between microfilarial intensity (mf/µL) and plumage quality (PC1). A significant negative correlation was observed (rs = −0.58, *P* < 0.001), indicating that higher parasite loads are associated with lower plumage quality scores. Individuals with higher PC1 values were predominantly found among those with low microfilarial intensity. The dataset includes 148 breeding males.

The robust linear models (RLM) further revealed that integrated plumage quality (PC1, accounting for 80.3% of total variation) was profoundly influenced by group status (F₃,₁₄₂ = 312.44, *P* < 0.0001, ηp² = 0.86). Sampling time had no significant influence on these scores (*P* = 0.39, ηp² = 0.03), confirming signal stability over the study period. Pairwise contrasts indicated significant differences among groups (Table 4; Fig 2A). While the healthy baseline remained unaffected by moult (NN vs. NM: *P* = 0.78, d = 0.08), substantial reductions were observed for the primary infection cost (NN vs. IN: d = 4.12) and the cost of infection during feather synthesis (NM vs. IM: d = 3.03). The cumulative stress of concurrent infection and moult resulted in a pronounced reduction in signal quality compared to the healthy baseline (NN vs. IM: d = 2.95, *P* < 0.0001). This statistical decline in PC1 was reflected by a marked reduction in hue, shifting from 55.1 ± 0.30° in healthy birds to 51.6 ± 0.30° in the synergistic group, alongside a notable decrease in chroma (0.49 ± 0.01) compared to the isolated infection group. Furthermore, the ornamental phenotype was strongly intensity-dependent; microfilarial intensity was negatively correlated with PC1 scores (r_s_ = −0.58, *P* < 0.001). Individuals with the highest parasite burdens exhibited the most pronounced reductions in signal quality (Fig 2B), with raw metrics indicating that high-intensity infections constrain the expression of vibrant yellow hue (shifting toward a duller 49.4–51.6° range) and reduced color purity compared to healthy breeding males (Table 1). Together, these findings suggest that filarial infection and concurrent moult drive a variable dynamic between intracellular redox status and metabolic demand throughout the reproductive cycle, where the cumulative cost of parasitism ultimately constrains the phenotypic expression of sexual signals.

**Table 4:** Statistical modeling of ornamental signaling quality and integrated plumage quality (PC1) in breeding male village weavers. Results of Robust Linear Models (RLM) evaluating the impact of experimental stressors and sampling time on plumage quality, followed by post hoc pairwise contrasts and Spearman’s rank correlation (rs) for parasite intensity-dependence (in bold). Sampling time was included as a fixed factor but was found to have no significant influence on integrated plumage quality (PC1; P = 0.39, ηp2 = 0.03), indicating that ornamental signaling remained stable across the study period.

Analysis Level	Source/ Contrast	Estimate/r_s_	*P*	Effect Size (ηp2/d)
Global Model	Plumage Quality (PC1)	(80.3% Var)	—	—
(RLM)	Group (Stressors)	—	<0.0001	0.86
Pairwise	Healthy Baseline (NN vs. NM)	0.12	0.78	0.08
Comparisons	Infection Effect (NN vs. IN)	−5.82	<0.0001	4.12
(PC1)	Isolating Cost (NM vs. IM)	−3.53	<0.0001	3.03
	Interaction (IN vs. IM)	2.41	<0.0001	1.84
	Cumulative Stress (NN vs. IM)	−3.41	<0.0001	2.95
**Mechanism**	**Parasite Load (r**_**s**_)	**−0.58**	**< 0.001**	**—**

## Discussion

In this study, while our findings are consistent with predictions regarding alterations in energy allocation, these associations should be interpreted with caution as they do not establish direct causality. Although our data demonstrate an association between microfilarial infection and a disrupted physiological state, the observational nature of this research means that the influence of confounding ecological factors cannot be fully excluded. Nonetheless, comparisons across infection statuses suggest a potential physiological trade-off, in which internal resource allocation may be associated with variation in external ornament expression [7,60].

In accordance with metabolic regulation theory, our results suggest that circulating glucose functions as a dynamic indicator of metabolic fuel mobilization rather than a static measure of energy reserves [61,62]. While circulating glucose provides a high-resolution snapshot of an individual’s immediate bioenergetic status, it reflects short-term physiological dynamics rather than long-term energetic stores such as fat or muscle protein. In the context of the village weaver’s breeding season, where immune activation and moult costs converge, fluctuations in glucose may indicate whether an individual can effectively mobilize energy or is experiencing energetic limitation. The elevated glucose levels observed in the infected non-moulting group (IN) are consistent with a transient mobilization of energy, potentially mediated by stress-related hormonal responses, to meet the immediate costs of immune activation [63]. In contrast, the lower circulating glucose levels observed in the infected moulting group (IM) may reflect a reduced capacity to sustain such compensatory responses under concurrent physiological demands, suggesting reduced capacity to maintain glucose homeostasis under combined physiological constraints [64,65] (Table 1).

The negative correlation between microfilarial intensity and the GSH:GSSG ratio (Table 2) further supports the interpretation of a trade-off between oxidative balance and immune function. Because glutathione plays a central role in both antioxidant defense and pigment-related biochemical processes, its depletion in infected individuals is consistent with a systemic trade-off rather than direct evidence of causation. It is likely that individuals capable of maintaining higher redox balance under infection pressure are better able to support the antioxidant demands associated with producing high-quality carotenoid-based signals (PC1). Correspondingly, reduced plumage quality in infected groups (IN and IM) may reflect underlying physiological constraints [66,67], consistent with carotenoid-based ornaments functioning as condition-dependent signals [68, 69].

In the absence of parasites (NN group), individuals exhibited relatively high antioxidant capacity and stable metabolic fuel availability, consistent with a homeostatic condition capable of supporting the energetic and nutritional requirements of plumage production (Table 1). During moult alone (NM group), these physiological markers remained relatively stable, suggesting that healthy individuals can buffer the costs of feather synthesis without substantial disruption to redox balance or metabolic regulation. In contrast, microfilarial infection appears to be associated with a shift in physiological allocation. In the infected non-moulting group (IN), reduced antioxidant ratio alongside elevated glucose suggests resource allocation toward immune function (Table 1), consistent with prior evidence of parasite-induced oxidative costs [61].

These internal physiological changes are also reflected in plumage coloration. The observed shift in hue in infected individuals may indicate altered carotenoid allocation, where pigments are preferentially utilized for physiological functions such as antioxidant defense rather than feather deposition [62]. Because pigment production and modification are closely linked to cellular redox processes, disruption to redox balance may constrain these pathways. Under such conditions, variation in plumage quality likely reflects limitations in pigment processing capacity rather than direct infection effects alone.

This pattern is most evident in the infected moulting group (IM), where individuals show lower circulating glucose relative to infected non-moulting birds (IN), indicating reduced metabolic flexibility under concurrent physiological demands (Table 1). Collectively, these observations indicate that infection and moult are associated with altered metabolic regulation, and that group differences reflect statistically supported Infection × Moult interaction effects estimated in the factorial model, rather than nonlinear or synergistic physiological processes.

Supporting this interpretation, regression analyses indicate that transient parasite dynamics are partially decoupled from physiological state (Table 2). Although microfilarial infection exhibit circadian periodicity and variation in prevalence [43,63], the absence of a sampling time effect on glucose and GSH:GSSG suggests that physiological costs are relatively stable across sampling periods. Pairwise contrasts further indicate that infected groups differ consistently in metabolic and antioxidant state, with IN showing elevated glucose but reduced antioxidant capacity (Fig 1A, C), consistent with compensatory physiological adjustment rather than enhanced condition.

The negative relationship between parasite intensity and antioxidant status (Fig 1B) further indicates that increasing parasite burden is associated with reduced redox balance. This is consistent with previous work on blood parasites, where infection is associated with increased oxidative challenge and reduced physiological buffering capacity [64]. Importantly, the interaction between infection and moult reflects additive effects captured by the factorial model interaction term, indicating that combined exposure modifies effect magnitude without requiring nonlinear interpretation.

The village weaver ornamental phenotype appears sensitive to these physiological changes. Reduced pigment quality in infected individuals is consistent with antioxidant allocation trade-offs under physiological stress. This interpretation is supported by depletion of the GSH:GSSG ratio across infected groups (Table 1) and aligns with findings in other species, such as the Great Tit (Parus major), where plumage coloration reflects physiological condition [65]. The decline in integrated plumage quality in infected groups indicates reduced expression of ornamentation associated with infection status rather than approaching physiological limits. Increasing parasite intensity is associated with reduced plumage quality (Fig 2B), supporting the hypothesis that carotenoid-based coloration reflects variation in physiological condition.

While our results focus on oxidative balance and plumage quality, they should be interpreted within a broader physiological context established in previous studies of this population. Across multiple studies in Village Weavers (Ploceus cucullatus), microfilarial infection has been consistently associated with energetic and physiological costs, including altered glucose and lipid metabolism, increased ketone production, and reduced body condition during energetically demanding periods [6,67]. Additional work has shown consistent associations between infection and antioxidant enzyme activity and stress physiology [10,68], reinforcing the interpretation that parasitism affects energetic allocation rather than a single physiological pathway. Collectively, these studies indicate that moult and breeding status modulate the magnitude of infection-related physiological effects, emphasizing life-history context in host–parasite interactions. However, because body mass and systemic condition indices were not measured in this study, these findings are not directly modeled here and should be interpreted as complementary contextual evidence.

## Conclusion

Although capture-based studies are valuable for understanding host–parasite interactions, our results suggest that single-point sampling primarily captures a snapshot of metabolic demand. The stability of key biomarkers across sampling times indicates that the GSH:GSSG ratio and circulating glucose are useful indicators of current physiological state. The transition from the relatively compensated condition observed in isolated infection to the more constrained state under combined stress may reflect a threshold in metabolic regulation, where the demands of immune activation and feather synthesis approach the limits of physiological capacity. This shift is associated with reductions in plumage hue, brightness, and chroma, supporting the hypothesis that village weaver plumage may function as an honest indicator of an individual’s capacity to manage simultaneous physiological challenges.

### Limitations

While our results provide evidence of an association between microfilarial infection and sexual signaling, several limitations should be considered. As an observational study, these findings do not establish causality, and other ecological factors, such as diet or co-infections, may also influence carotenoid availability and redox balance. In addition, the absence of longitudinal data limits our ability to assess long-term fitness consequences, including survival and reproductive success. Future research should incorporate experimental approaches, such as antioxidant supplementation, to test the mechanistic basis of these trade-offs. Expanding analyses to include females, individuals of different age classes, and multiple species would further clarify the generality of these patterns. Longitudinal studies are also needed to determine whether the observed physiological constraints are associated with reduced social dominance or mating success. Finally, integrating molecular approaches, such as gene expression analyses related to carotenoid processing, would provide deeper insight into the mechanisms linking physiological stress and ornamental trait expression in wild populations.

## Supporting information

S1 Dataset Supporting Information. The dataset used in this study is provided in CSV format.
(CSV)

S1 File Supporting Information – Compressed/ZIP File Archive. Compressed archive containing supplementary materials associated with this study.
(XLSX)
